# Supplementary material for: Transcriptomic Insights into Mechanisms of Early Seed Maturation in the Garden Pea (Pisum sativum L.)
Source: Cells. 2020 Mar 23;9(3):779. doi: 10.3390/cells9030779 (PMC7140803; doi:10.3390/cells9030779)

Sample SOM on module data (dmap)

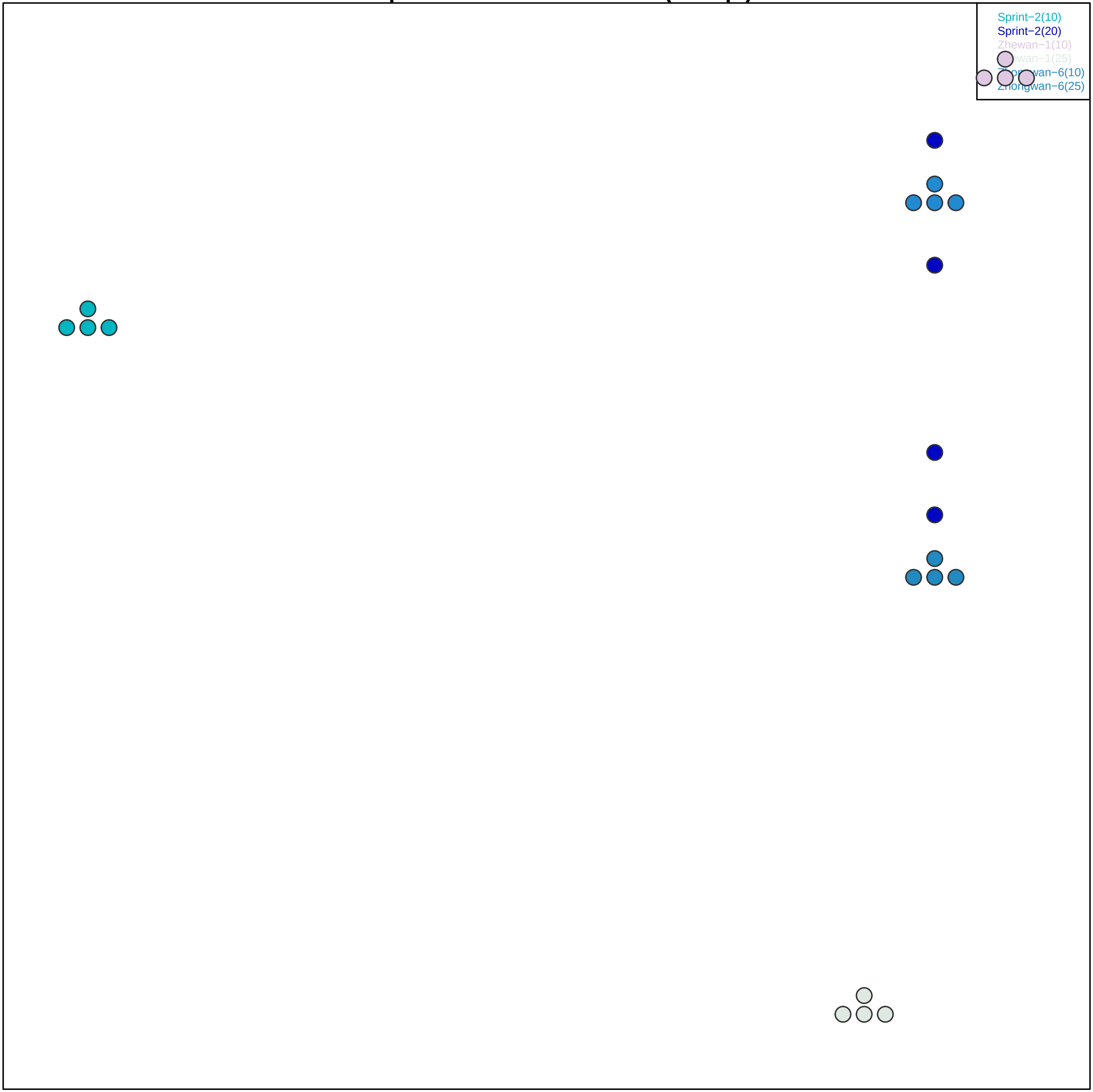

Sample SOM on module data (dmap)

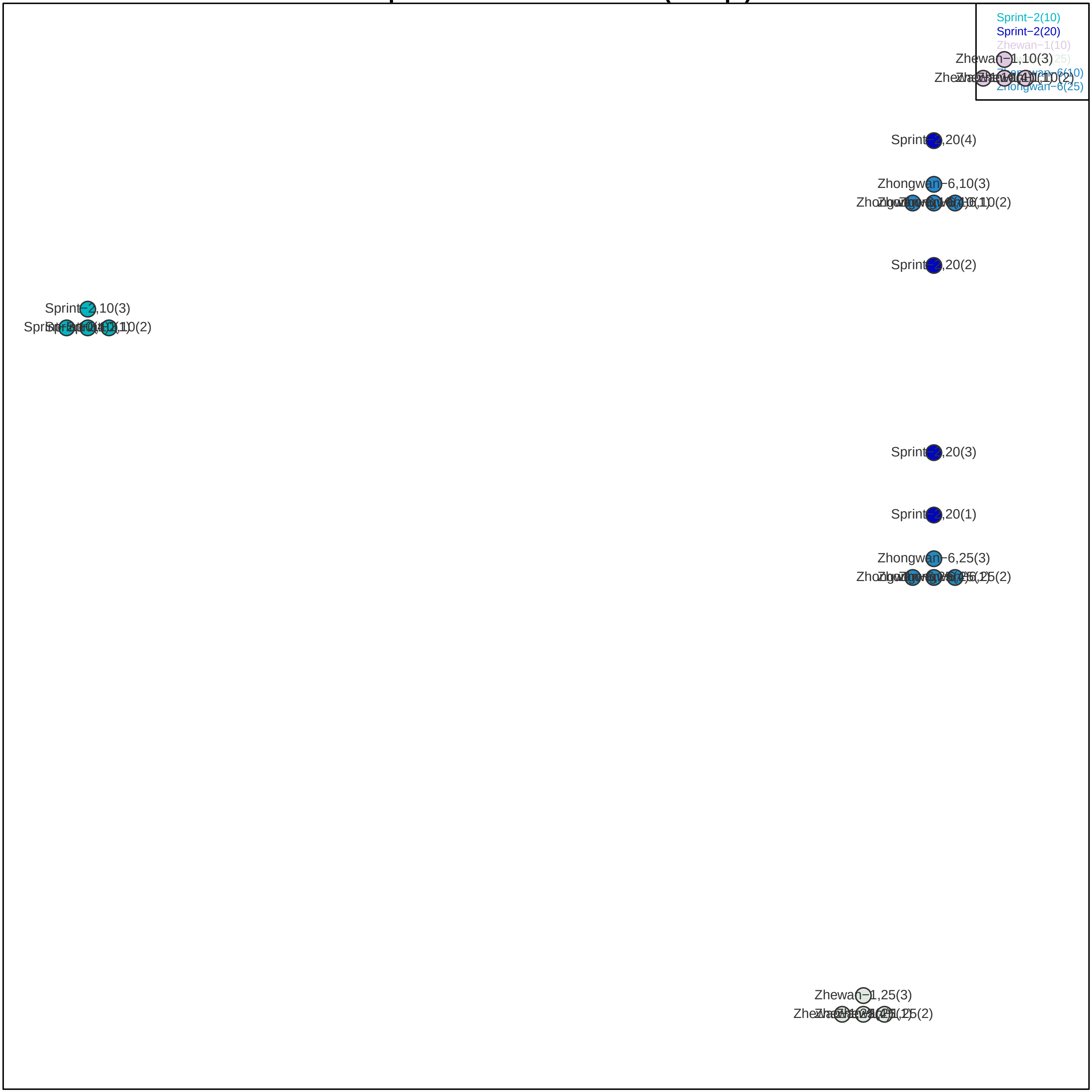

Second level SOM, group outlines

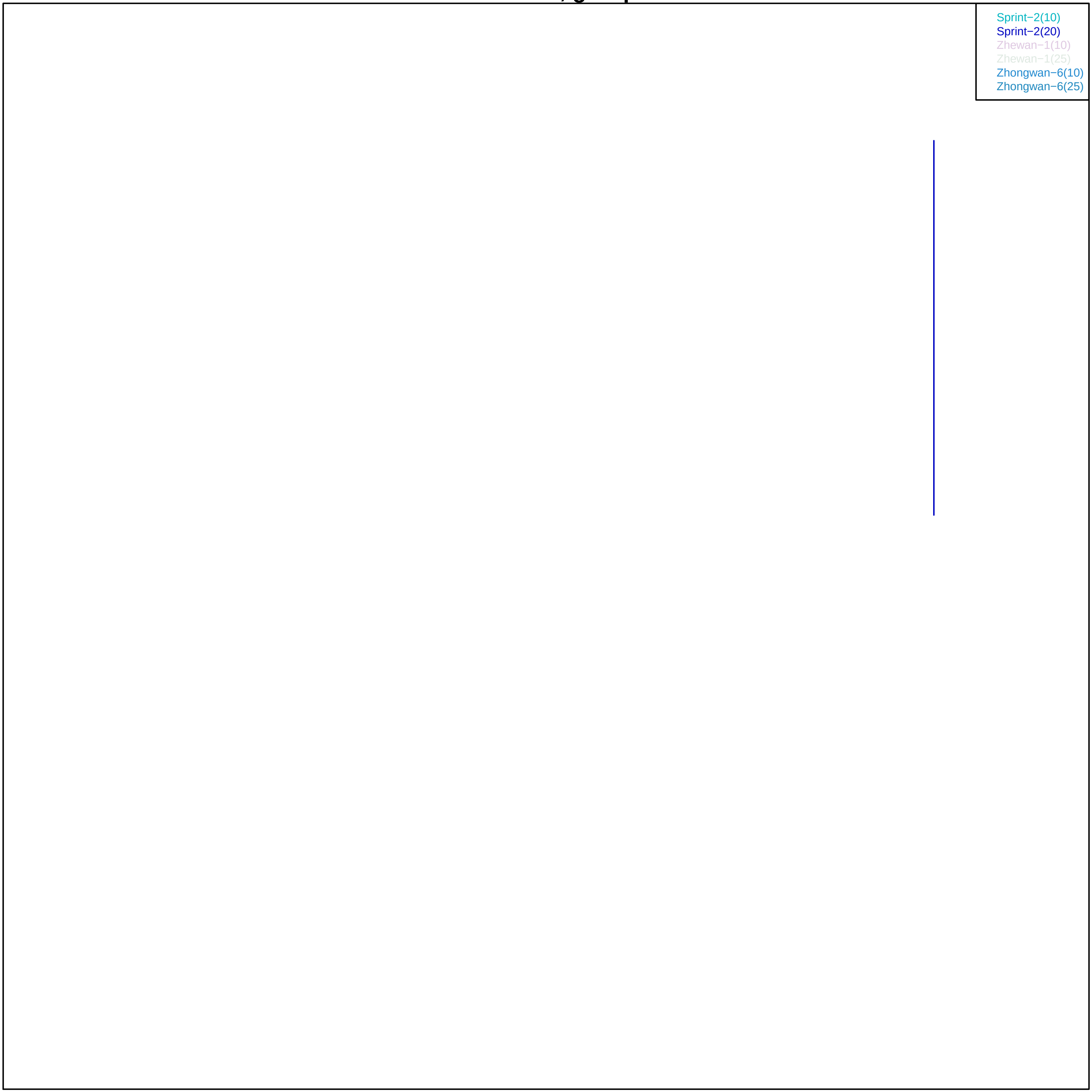

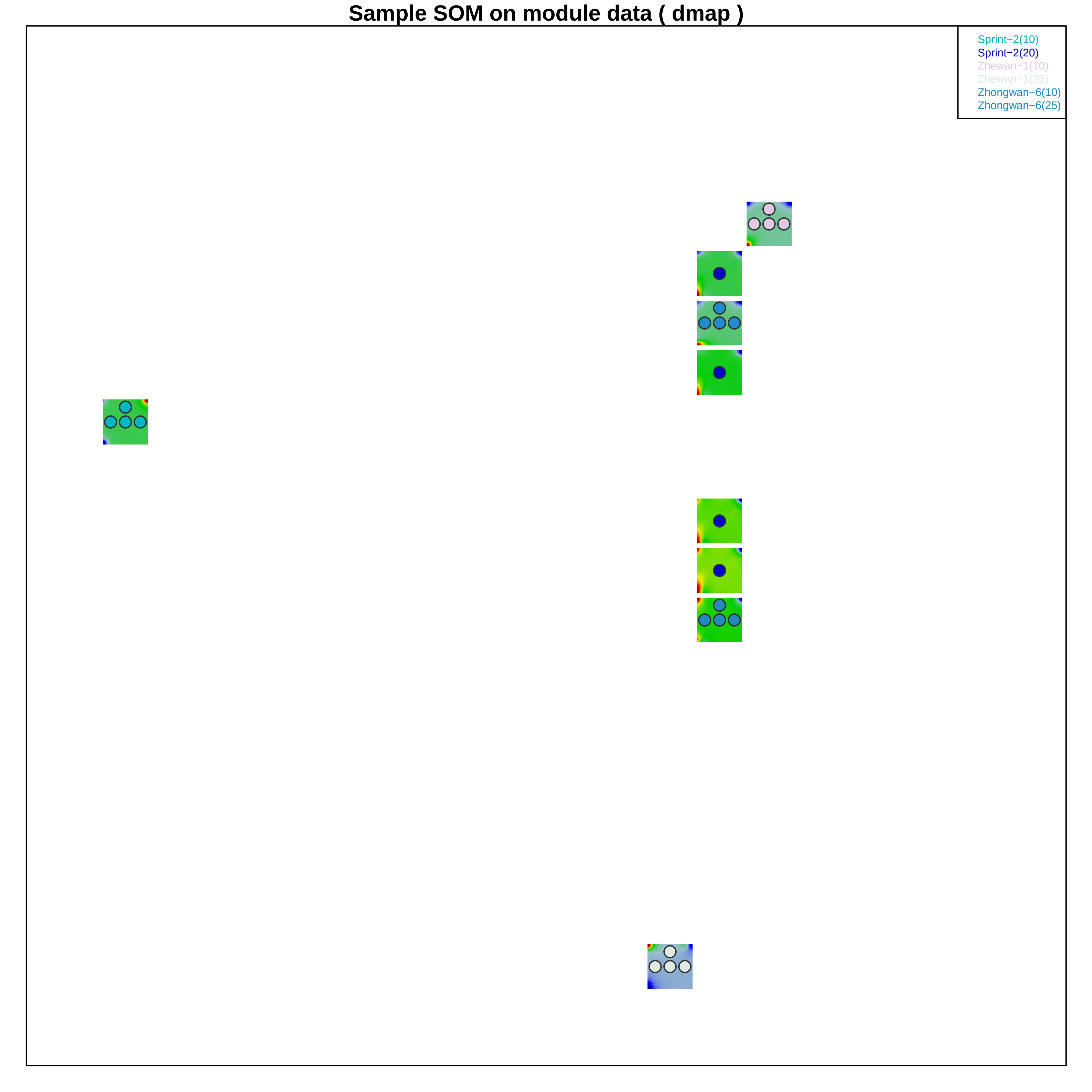
Sample SOM on module data (dmap)
Sprint−2(10)
Sprint−2(20)
Zhewan−1(10)
Zhewan−1(25)
Zhongwan−6(10)
Zhongwan−6(25)

Sample SOM on metagene data

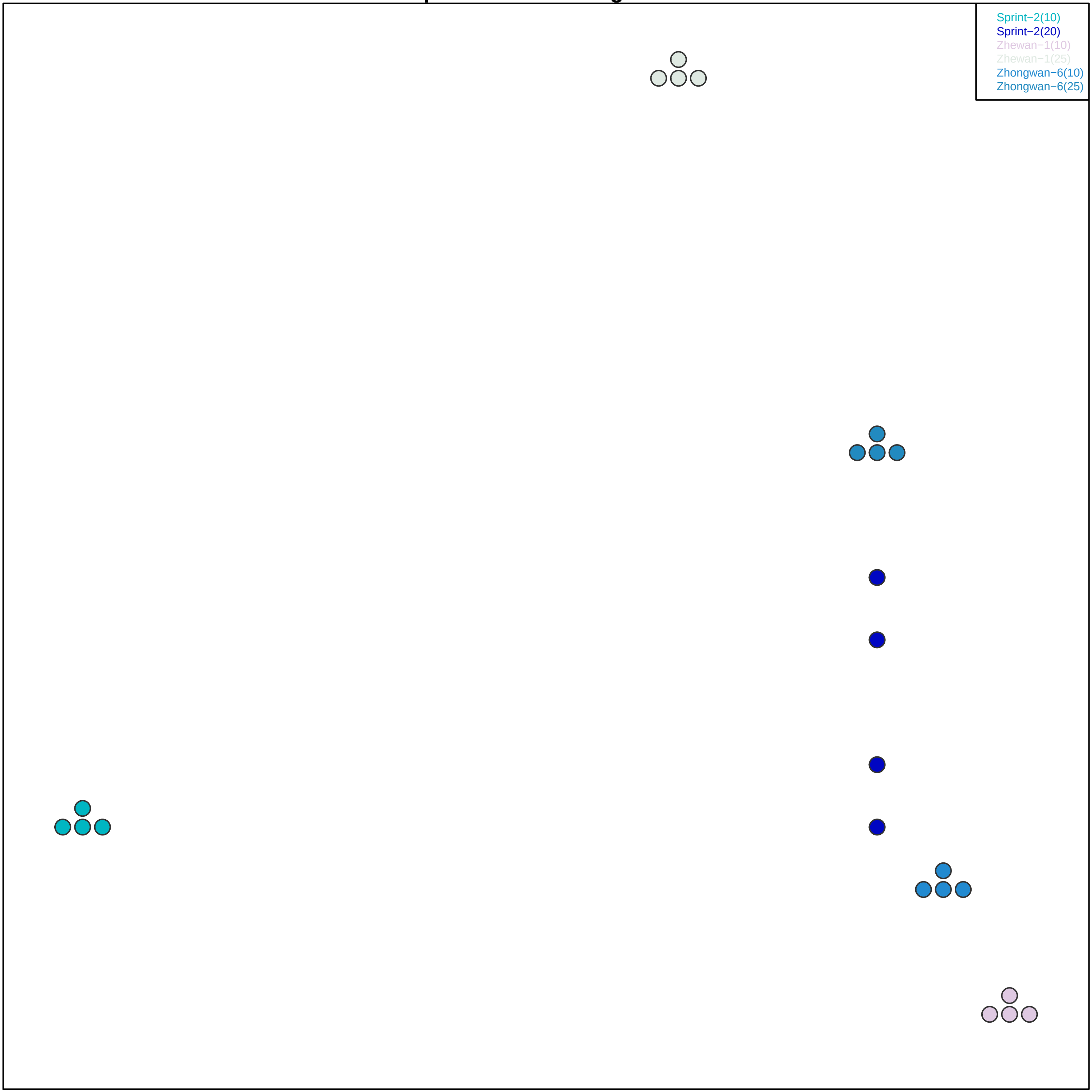

Sample SOM on metagene data

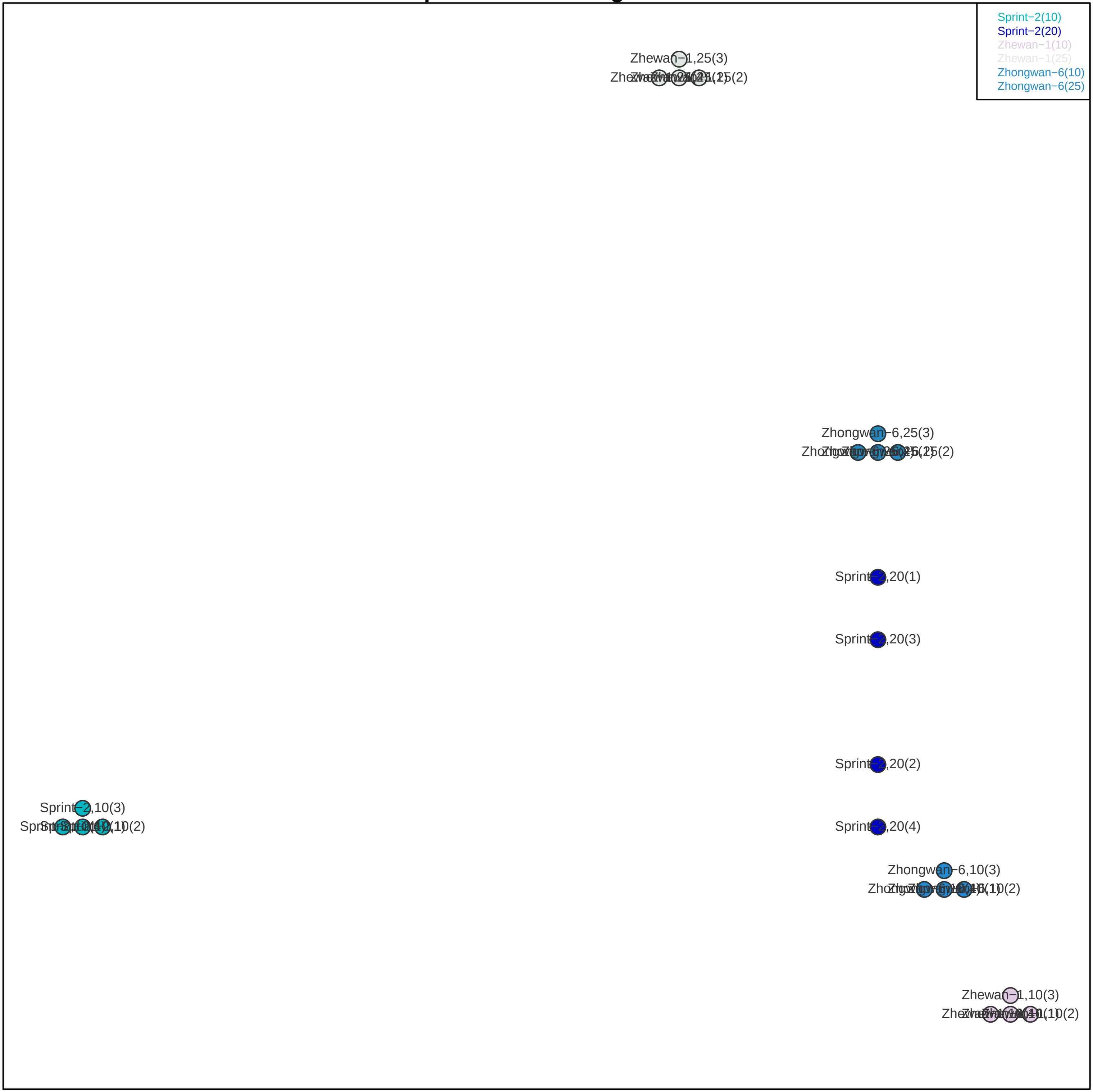

Second level SOM, group outlines

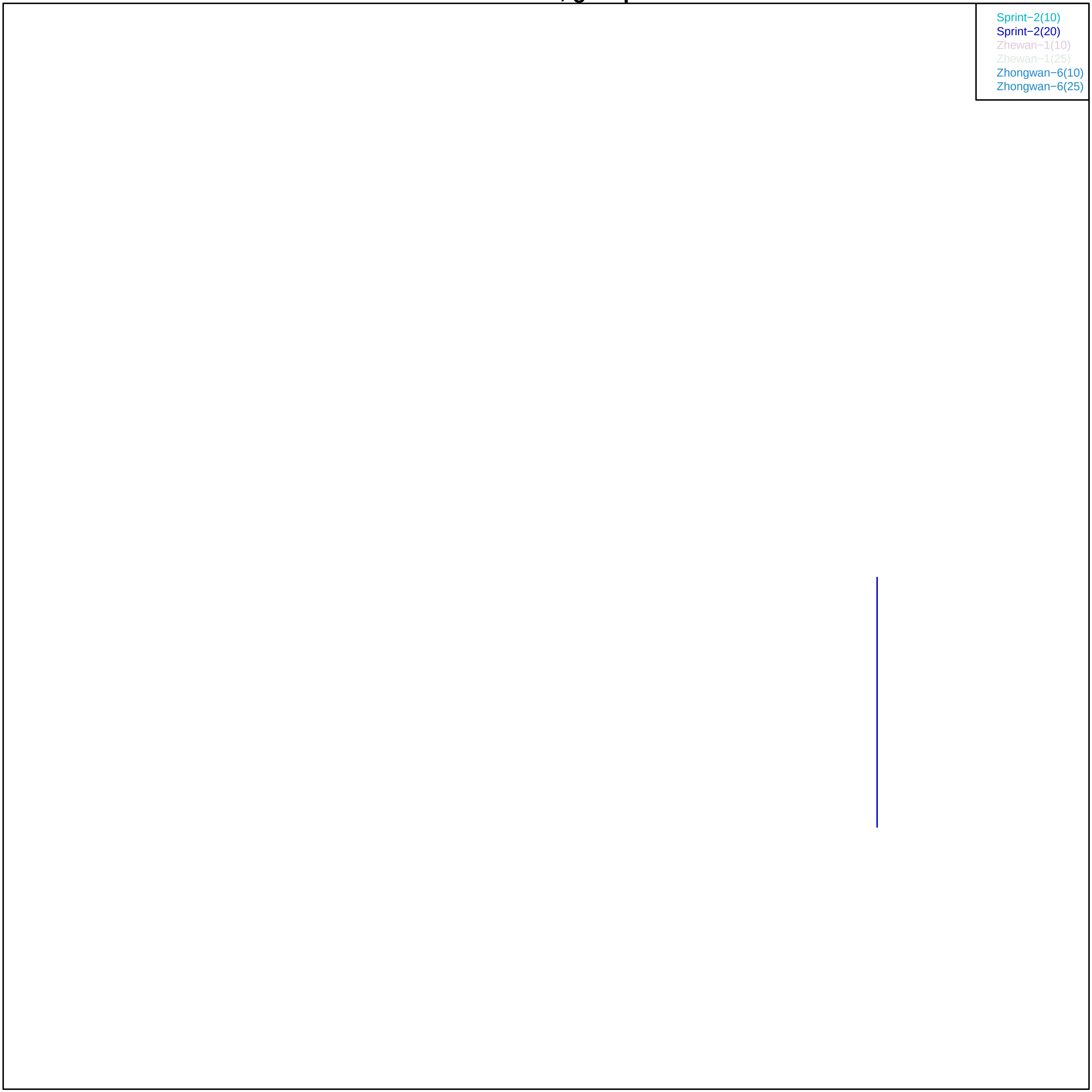

Sample SOM on metagene data

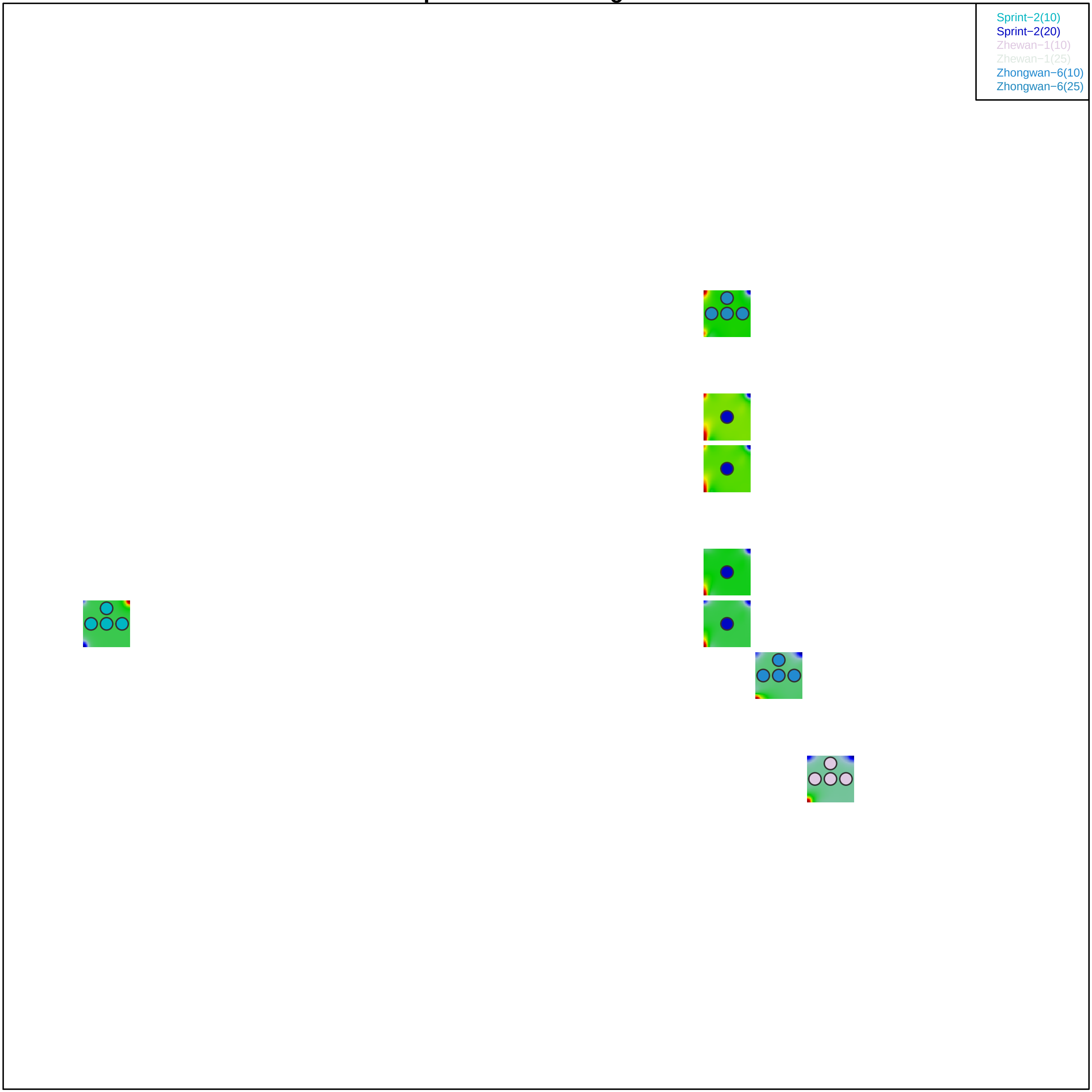

Supplement: Supplementary file 1 [file cells-09-00779-s001.zip › Supplementary materials/FileS2.zip!/FileS1/Sample Similarity Analysis/Sample SOM.pdf]
